# On a Substitute for Alcohol in Making Anatomical Preparations

**Published:** 1817-03

**Authors:** William Cooke

**Affiliations:** Surgeon.


*For the London Medical and Physical Journal.*

### *On a Substitute for Alcohol in making Anatomical Preparations;* by Mr. William Cooke, Surgeon.

WITH a practitioner in medicine, anatomy and physiology should always be directed to the improvement of pathology.

There are, unhappily, great impediments to the cultivation of this part of medical science, derived chiefly from the prejudices of mankind against what is accounted a mutilation of the body; and no arguments will generally avail to counteract this repugnance to opening the *dead*, but those that are dictated by a mind deeply impressed with the advantages which accrue to the *living* from such investigations.

Whenever practicable, the preservation of specimens of natural and morbid structure contributes greatly to enhance their value, by making them not only objects of occasional reference to the primary possessor, but, when he ceases to exist, they are handed down, and perpetuate instruction to posterity.

The expence of glass and spirits has deterred most practitioners from accumulating specimens of this nature, and therefore every suggestion designed to remove the obstacle is worthy of consideration.

During some years, I have been accustomed to preserve diseased parts, and, although on a very circumscribed plan, it has been sufficiently extensive to enable me to judge tolerably well respecting the comparative effects of different preserving media.

The object of this paper is to lay before the profession a few recent experiments made with a solution of the *solid* salt, a purer muriate of soda than the salt of commerce. I shall detail the facts as accurately as I can; for, notwithstanding the success of these experiments has been such as to induce me to give them publicity, I neither wish to excite unwarrantable expectations, nor to pledge myself for the result of more extended essays. It is but equitable, however, to claim the usual concession to first attempts, especially as it was requisite to deviate, in various circumstances, from the common methods of preservation.

Last summer I had the pleasure of introducing Phillips London, Esq. proprietor of the British solid salt, to the Hunterian Musæum. Whilst viewing that splendid exhibition, Mr. London suggested the trial of his salt, which had been found much more effectual in *pickling* meat than other salt; and solutions of which, he had ascertained, would preserve serve the natural appearance of fish. He soon afterwards politely furnished me with a quantity for that purpose, of which the dates and results are as follow:—

| No. | 1816 June | *Time of Preservation.* | *Present appearance.* |
|---|---|---|---|
| 1 | 18 | A portion of stomach, from a young man poisoned by arsenic. | The inflamed condition of the stomach, and enlargement of the mucous glands, the same as when taken from the body, but within about the last month a few gaseous bubbles have escaped. The transparency of the fluid continues, although it is not perfectly bright. |
| 2 | do. | A small hydrocele, injected with size and vermillion; but the injection did not run well, and the testicle was a little soiled in handling. | In good preservation, one small bubble now resting at the top—fluid clear and colourless; but, when agitated, a few very small flocculent substances are seen in it. The colour of the injection unchanged. |
| 3 | do. | A portion of duodenum, to shew the valvulæ conniventes, and some scrofulous mesenteric glands. | In excellent preservafion—the fluid bright, and both the natural and morbid appearance of the parts well retained. |
| 4 | 24 | Part of the stomach of a young man poisoned by opium. | Fully retains its morbid character; but some bubbles of gas have escaped, and are entangled at the top between the talc and specimen—liquid colourless and bright. |
| 5 | do. | Large portion of the heart, to exhibit an open and reticulated foramen ovale in the adult. | In excellent preservation, but appears somewhat hardened. The fluid colourless and transparent, although not so bright as some others. |
| 6 | do. | Some tuberculated intestine. | Well preserved. |
| | Aug. | | |
| 7 | 20 | Uterus and appendages. | In perfect preservation—fluid colourless and transparent. |
| | Sept. | | |
| 8 | 10 | A portion of stomach acted on by the gastric juice, and imperfectly injected with size and King's yellow. | As perfect and bright as possible—colour of the injection unimpaired. |

The

The eye, in different states, has been in the solution from the earliest periods of these experiments; but, not having by me any proper sized bottles, they were not put up. One, however, which has been in the fluid upwards of seven months, to ascertain its effects on the vitreous humour and lens, now stands before me. It retains its natural figure and consistence. The membrana hyaloidea is only affected in its transparency just enough to distinguish it from the circumjacent fluid. The lens has a yellowish opake circumference, yet sufficiently penetrable to exhibit a transparent centre. By the section of another lens, I find these parts retain their usual dissimilarity of structure,—the *outer* being softer, and the *inner* acquiring, by progressive laminæ, a horny density. The capsule of the lens is exhibited with considerable beauty, and is uniformly separated from the *proper* membrane of the lens by the interposition of a transparent aqueous humour. When the lens had been removed, the posterior part of the capsule retained its proper form and texture, but the anterior became like transparent horn. The eyes were those of the calf.

Some fœtuses, at different periods of conception, have been put up; but, as they had been in spirits of turpentine before being given to me by a medical friend, they do not afford any legitimate inferences, although well preserved.

An aphrodita, and some other small specimens, have been put up; and, deducting something for a little inattention to neatness, they correspond, in general, with the preceding.

About two months ago, I removed a pendulous tumour from the labium pudendi, which, during the pregnancy of the lady, always became œdematous, and as large as the feet; but contracted about one half after parturition. It was of very loose cellular structure, and, after remaining about a week in the solution at saturation, it corrugated, and greatly diminished. When it had been in water for twenty-four hours, it re-assumed its first appearance, and I put it into a weak spirit.

Whether diminishing the strength of the brine would have succeeded, I did not attempt to ascertain.

I have had mere fleshy parts in a covered pan for some months, which continue unchanged.

It remains for me now to relate those particulars which have appeared necessary preliminaries to success.

The solution I keep by me at saturation, which contains about 28 per cent. of salt. Mr. London has obliged me with two glass floats, one of which rises to the surface at saturation, and indicate s. g. about 1.206; the other rises at 1.160. These afford considerable facility in ascertaining the strength.

By

By keeping the solution at its greatest strength, it can be reduced to any suitable degree.

When I put up a preparation in this solution, I add some very clear water, in the proportion of half an ounce to a pint. This is done to guard against crystallization; for, although even at saturation no crystals can form without evaporation, by adopting the above precautionary measure, a greater length of time would be requisite before inconvenience could result, should the escape of water not be completely prevented.

It seems best not to macerate the substance so long in water as is usually done for spirit; but, after having been in water, frequently changed from one to three or four days, it should be placed in some common receptacle, containing solution for some days or weeks as may be most convenient. A glazed earthen pan with cover answers very well. The specimen, by retaining some of the blood, looks more natural; and, by an earlier removal from the water, future decomposition is more effectually suspended.

On being first put into the solution, animal matters float; but, in twenty-four hours or more, according to their nature, they sink. Where, however, they have not sufficient gravity, no deformity arises from their suspending small glass bodies. The texture of some animals is such, that they will never sink unassisted; and, whenever this happens, threads over the rim may be dispensed with.

One objection has been suggested by high professional authority, viz. that, *in three years*, the preparation will shrivel.—I must adopt Bacon's maxim, and say, *fiât experimentum*. At present, no approximation to this state is visible; and, if I have a correct idea of the chemical action of muriate of soda on animal matters when *perfectly* excluded from the atmosphere, such a contraction is highly improbable.

The usual method of closing with bladder and lead is ineligible, for, when the bladder becomes saturated, the salt in time penetrates the coverings of the rim, and crystallizes on the bottle; and, independently of this exudation, the lead would gradually be decomposed by the solution. I have tried several modifications of this plan, but all seem uncertain or troublesome.

I now adopt a piece of common window glass, cut to the size and shape of the bottle, and fixed either by resin or putty. Putty answers tolerably well where there are no threads to sustain the specimen, but, where there are, they are liable to slide in, or to act as siphons, till it dries. Resin succeeds succeeds in all cases so well, as to justify a decided preference. Having wiped the edge of the bottle dry, I spread on it some melted resin, softened by a few drops of oil, and lay on it the glass. By holding over it a spatula considerably heated, the cement liquifies, and the glass fits with the utmost accuracy. After smoothing the resin, I have covered it in some instances with black varnish, without further trouble; but, as an additional security, it is preferable to besmear the edge with some glue, and lay over the top a piece of moistened bladder.

Persons are accustomed to lift their specimens by the rim, and, if the glass have any angular projections, and were unprotected by bladder, it might occasionally be raised; it is proper, therefore, to keep it a little within the circumference of the *round*. The center of the bladder may be removed, so as to leave the largest part of the top diaphanous.

The description of manual operations, in which there are several minute objects for attention, is apt to convey an improper idea of the trouble attending them; whatever may be inferred from *reading* the above, I am persuaded those who *practise* it, will find it more easy and neater than the method with half-putrid bladders and lead.

No inconvenience has resulted when the diameter of the glass did not exceed two or three inches; but, at four or five inches, it has sometimes cracked, after having been finished some weeks. Whether this has arisen from variation in the pressure, or bad quality of the glass from not being well annealed, I am not satisfied. In two instances, I have used the *eye* of the glass, having its convexity central. Each of these has a diameter of four inches, and has been up four or five months; one remains perfect, the other, soon after being fixed, cracked about half an inch at two opposite points, but continues in *statu quo*, and is as effectual as at first, nor would the cracks probably have been discovered had I not sought for them. The convex top, I think, improves the appearance of the bottle.

Where bubbles have yet appeared in the specimens, maceration had been continued longer than is advisable, and it is suspected decomposition had commenced before they were put into the solution.

As the expence of this salt is very trivial, I intended to have ascertained whether it might not be subservient to important purposes on a larger scale, but circumstances have hitherto prevented. My design was to have procured a small adult subject, to have divided it in such a manner that all the important parts should remain in their natural posi-

 tion, tion, and so place them in a covered tub of the solution. If this succeed, a practitioner might always have in his possession a subject for anatomical research; for, even, should it harden, maceration in water for twelve or twenty-four hours would render it sufficiently soft for dissection, and it might easily be re-salted. The proper strength of the fluid would be kept up without inconvenience, and almost without expence. Should evaporation happen, the addition of water will prevent crystallization; and if, by frequent examination and maceration in water, the strength be reduced, only the addition of a proportionate quantity of this muriate of soda would be needful. I cannot conceive that any thing more can be requisite to ensure success than carefully to keep the animal substance beneath the surface of the brine.

In the event of success, the plan may be of incalculable advantage to country practitioners; for, however valuable dry preparations may be to illustrate the vascular system, no correct knowledge can be gained of anatomical structure, but by observing all the parts in their natural and relative positions. The conveyance of the salt to any part of the country would be effected with the utmost facility.

Some attention to the dissecting instruments will be necessary to keep them from being corroded.

Whether the common muriate of soda would avail, I cannot determine; but, as the solid salt is more effective in the preservation of meat, it is reasonable to infer its superiority for anatomical purposes. The operation by which it is distinguished in its preparation from the common salt deprives it totally of the muriate of magnesia, which, I am informed, promotes putrefaction.

It must be understood, that the plan is chiefly recommended for those to whom economy is important; at the same time, it seems applicable to some purposes for which the volatility of spirit renders it unfit.

It did not concur with my object, to sanction these experiments with the approbation of distinguished characters, yet, independently of the personal honour I feel in being permitted to introduce the name of Sir Joseph Banks, the permission derives importance from the influence which he has long illustriously diffused by a most liberal cultivation of the arts and sciences.

At the request of Mr. London, and by a previous arrangement with Sir Joseph, I laid before, and left with him, the specimen No. 1, and that of the eye alluded to above, and waited on him a few days since to ascertain the result. He said they had been shewn to several eminent anatomists who resorted thither, and, in general, the plan was considered

 novel,—

novel,—much approved,—and deemed highly worthy of an extended trial. The specimen of the eye, he said, was especially admired.

*Great Prescot-street;*

*Feb.* 1, 1817.

---

*For the London Medical and Physical Journal.*

