# Errata

**Published:** 1817-04

**Authors:** 


					Page ^82, 1, 17, read fist forfeit.?Ditto, I. 9) mart for mere.

				

